# Ex-situ X-ray computed tomography data for a non-crimp fabric based glass fibre composite under fatigue loading

**DOI:** 10.1016/j.dib.2017.10.074

**Published:** 2017-11-03

**Authors:** Kristine M. Jespersen, Lars P. Mikkelsen

**Affiliations:** Department of Wind Energy, Section of Composites and Materials Mechanics, Technical University of Denmark, Risø Campus, 4000 Roskilde, Denmark

## Abstract

The data published with this article are high resolution X-ray computed tomography (CT) data obtained during an ex-situ fatigue test of a coupon test specimen made from a non-crimp fabric based glass fibre composite similar to those used for wind turbine blades. The fatigue test was interrupted four times for X-ray CT examination during the fatigue life of the considered specimen. All the X-ray CT experiments were performed in the region where unidirectional fibre fractures first became visible, and thereby include the damage progression in 3D in this specific region during fatigue loading of the specimen.

**Specifications Table**

**Table:** 

Subject area	*Physics*
More specific subject area	*Fibre composites, Damage mechanics*
Type of data	*Image (X-ray computed tomography data sets)*
How data was acquired	*Zeiss Xradia Versa 520 (X-ray CT)*
Data format	*Raw, Reconstructed*
Experimental factors	*Interrupted tension-tension fatigue test (R=0.1)*
Experimental features	*High resolution X-ray CT scans performed in the same region after each interruption point of the fatigue test*
Data source location	*Roskilde, Denmark*
Data accessibility	The data is available online at: 10.5281/zenodo.845707

**Value of the data**

• The data makes it possible to observe damage progression inside the specimen, and can be used for further establishment of automatic 3D visualisation methods, which could enhance the understanding of the damage progression mechanisms even further.
• The data can serve as a comparison base and as initial knowledge for modelling of the damage progression.
• The detailed damage mechanisms in the data sets can be explored further if automatic image analysis methods are developed for quantification of the damage relative to the fibre and fibre bundle architecture.

## 1 Data

The data published here consist of four sets of X-ray CT data captured after each interruption point of a tension-tension fatigue test (47,300, 57,300, 67,300, and 77,300). For each interruption point, the raw projection data in the “.txrm” format and the reconstructed data in the “.tif” format along with relevant scan settings (labelled “info1” and “info2”) are provided. The “.txrm” format is the regular output format for the raw image data of the Zeiss Xradia Versa 520 system used for the experiments before reconstruction. In addition, a large field of view (LFOV) dataset and a video showing the 3D visualisation of uni-directional fibre fractures (Fig. 9 in [1]) was also included as a supplement to the ex-situ X-ray CT data*.*

## 2 Experimental design, materials and methods

### 2.1 Test specimen and fatigue testing

Ex-situ X-ray CT fatigue experiments were carried out on a 410 mm long butterfly shaped test specimen optimised for testing uni-directional (UD) fibre composites [2] with a 15 mm wide gauge section [1]. The material system considered was a glass fibre non-crimp fabric reinforced polyester composite with the layup [b/biaxial,b/0,b/0]_s_ where “b” refers to the supporting backing layer and “0” to the UD fibre bundles, which are stitched to the backing layer. The supporting backing layer is made from fibre bundles oriented in the directions 45°/90°/-45° and is significantly thinner than the UD layer of the fibre composite (see also [1]). The backing fibre bundles have a significantly larger spacing than the UD fibre bundles and in some regions cross over one another due to their lay-up.

The tension-tension fatigue test was carried out in load control with a stress ratio of *R*=0.1 at a test frequency of 5 Hz and an initial strain of *ϵ*=1%. Initially two static tests were performed to obtain the initial stiffness and thereby estimate the load corresponding to 1% strain of the specimen prior to the fatigue test. The strain was measured over a 25 mm length in the gauge section of the specimen using extensometers. The fatigue test was interrupted for X-ray CT examination after 47,300, 57,300, 67,300, and 77,300 load cycles followed by failure of the specimen. The last interruption point was close to final failure (see also [1]).

### 2.2 X-ray computed tomography

X-ray CT experiments were carried out after each interruption point of the fatigue test where the same region was scanned multiple times. To do so, the specimen was taken out of the fatigue testing machine and mounted in the X-ray CT scanner using a special holder making it easy to mount the specimen in the same way each time. After remounting the specimen in the X-ray CT scanner, the positioning was manually fine-tuned by comparing the 2D projection images from two sides of the specimen to those of the first interruption point. A 2000×2000 pixel detector with an optical magnification of 4× was used, and the scans were carried out with a binning of 2 resulting in 1000×1000 pixels in the projection images. The images were captured with a source to sample distance of 28 mm and a detector to sample distance of 35 mm resulting in a pixel size of 3 µm. The experiments were carried out using an accelerating voltage of 70 keV, and exposure time of 7 s. 4601 projections were captured during a full rotation of 360 degrees. For each data set there can be a slight variation in the settings, however the detailed settings for each of the four X-ray CT experiments can be found labelled by “info1” and “info2” in the data published with this article. To consider the same region of the specimen repeatedly.
